# The impact of the consistency evaluation policy of generic drugs on the integration of innovation chain and industrial chain in the pharmaceutical manufacturing industry

**DOI:** 10.3389/fpubh.2026.1791110

**Published:** 2026-03-18

**Authors:** Yanqing Xie, Wenjing Zhang

**Affiliations:** 1 Institute of Applied Economics, Shanghai Academy of Social Sciences, Shanghai, China; 2 Institute of Economics, Shanghai Academy of Social Sciences, Shanghai, China

**Keywords:** consistent evaluation policy of generic drugs, difference-in-differences model, industrial chain, innovation chain, pharmaceutical manufacturing industry

## Abstract

**Introduction:**

The Consistency Evaluation Policy of Generic Drugs is a major quality-oriented regulatory reform in China’s pharmaceutical manufacturing industry. Whether and how this policy facilitates the integration of the innovation chain and the industrial chain at the enterprise level remains insufficiently examined. This study evaluates the policy effect and investigates potential mechanisms.

**Methods:**

This study used panel data on A-share listed pharmaceutical enterprises from 2013 to 2023. Enterprises were treated as the micro-level carriers of both the innovation chain and the industrial chain, and a enterprise-level index was constructed to measure their integration. A difference-in-differences (DID) design was employed to estimate the impact of the Consistency Evaluation Policy of Generic Drugs. Mechanism analyses focused on government subsidies and market concentration, and heterogeneity was assessed by market demand and total factor productivity (TFP).

**Results:**

The Consistency Evaluation Policy of Generic Drugs significantly promoted the integration of the innovation chain and the industrial chain. Mechanism tests suggested that the effect operated through two channels: increased government subsidies and higher market concentration. The positive effect was stronger among enterprises facing larger market demand. Moreover, the effect was significant for enterprises with higher TFP, while it was not statistically significant for enterprises with lower TFP.

**Discussion:**

These findings suggest that policy implementation can be strengthened by (1) improving the depth and precision of the Consistency Evaluation Policy of Generic Drugs, (2) enhancing the targeting of government subsidies and supporting an appropriate degree of industry concentration where warranted, and (3) adopting differentiated guidance to stimulate enterprise vitality through multiple measures.

## 1 Introduction

According to data from the National Medical Products Administration, generic drugs account for approximately 95% of all drug approvals in China. However, gaps remain between some domestically produced generics and original drugs in terms of efficacy and safety. In addition, high-quality drug markets in some therapeutic areas are still dominated by original drugs. Low-quality generics may compromise treatment effectiveness and medication safety (1). They may also increase the overall healthcare burden and weaken public trust in drug quality, thereby raising medical insurance expenditures and reinforcing low-end competition in the pharmaceutical manufacturing industry. Therefore, in 2016, the former China Food and Drug Administration implemented the Consistency Evaluation Policy of Generic Drugs to ensure that evaluated generic drugs are fully consistent with originator drugs in terms of therapeutic efficacy and quality (2, 3). The United States and Japan have also conducted drug evaluation programs similar to China’s consistency evaluation (4, 5). However, China’s consistency evaluation additionally aims to promote the clinical substitution of generic drugs for originator drugs whose patent protection has expired. Therefore, China’s consistency evaluation is not only about enhancing quality at the technical level, but also aims to optimize the industrial structure of the pharmaceutical sector and reduce the burden on medical insurance. Ultimately, it is expected to shift the pharmaceutical industry from low-end manufacturing to innovative development. Nowadays, promoting the high-quality development of the pharmaceutical manufacturing industry has become a national strategic priority, with the core being the deep integration of the innovation chain and the industrial chain.

At present, the relevant research mainly focuses on three aspects: First, studies examine the economic and industrial effects of the Consistency Evaluation Policy of Generic Drugs. As a profound measure of supply-side reform, its impact extends well beyond the initial goal of ensuring the quality of generic drugs. By setting strict thresholds for quality and therapeutic efficacy, the policy forces the exit of outdated capacity and reshapes the competitive landscape. As a result, enterprises may increase research and development (R&D) investment (6) and upgrade industrial technologies. This, in turn, can strengthen applied research at the downstream end of the innovation chain and improve quality management at the upstream end of the industrial chain (7). Moreover, the Consistency Evaluation Policy of Generic Drugs guides key resources toward higher-quality enterprises. It also promotes closer collaboration among industry, universities, and research institutes, and supports the integration of research, development, and application. In addition, the policy generates new upstream demand in the industrial chain for high-end active pharmaceutical ingredients, advanced excipients, and precision equipment. Second, scholars study integration between the innovation chain and the industrial chain. Existing work often measures the degree of integration at the industrial or regional level (8, 9). Common approaches include the coupling coordination degree model and improved distance-based collaboration models (10, 11). The literature also identifies enabling factors. Talent, capital, and the digital economy can play bridging and catalytic roles in integrating the two chains (12, 13). These factors help connect critical links from basic research to industrialization. Third, research examines how industrial policies facilitate integration between the innovation chain and the industrial chain (14). Earlier studies emphasized the direct effects of policy tools on innovation inputs, often describing a one-way pathway from policy incentives to enterprises’ R&D behavior. More recent work pays closer attention to transmission mechanisms. A common view is that industrial policies can guide the agglomeration of capital and talent in specific segments through resource-allocation effects (15). This may promote stronger alignment between the two chains in terms of objectives, actors, and processes.

Existing studies provide an important foundation for this paper. However, research on the Generic Drug Consistency Evaluation policy has largely focused on its impact on individual aspects, such as industrial landscape and corporate R&D. There is a lack of systematic investigation that places this policy within the framework of innovation chain and industrial chain integration. Consequently, its deeper driving effects on the integration of the dual chains have not yet been fully revealed. In addition, prior research on two-chain integration frequently relies on macro-level panel data and focuses on regional or industry-level measures. Enterprise-level empirical evidence is comparatively limited. Yet enterprises are central actors in two-chain integration, and their strategic responses are important for understanding policy effectiveness. This micro-level mechanism therefore warrants further examination. Finally, although the literature on industrial-policy mechanisms has discussed intermediaries such as resource allocation and knowledge integration, more systematic work is needed to place policy tools, transmission channels, and two-chain integration within a unified analytical framework.

Compared with previous studies, this paper makes three contributions. First, it offers a relatively new perspective. The integration of innovation chain and industrial chain is a key pathway for the high-quality development in the pharmaceutical manufacturing industry. It is shaped not only by the level of human capital, but also by financial support and industrial ecology. By incorporating the Consistency Evaluation Policy of Generic Drugs into the analysis, this study provides a more comprehensive explanation of the factors associated with dual chains integration in the pharmaceutical manufacturing industry. Second, from the perspective of node enterprises, this study estimates the effect of the policy on dual chains integration and tests two mechanisms, namely government subsidies and market concentration. Third, this study examines the heterogeneity in the policy effect across different levels of market demand and enterprise total factor productivity (TFP). The results provide theoretical and empirical support for designing differentiated policies in the pharmaceutical manufacturing industry.

## 2 Theoretical analysis and research hypotheses

### 2.1 Integration of consistency evaluation policy with the innovation chain and industrial chain in the pharmaceutical manufacturing industry

Reverse pull of consistency evaluation on the innovation chain. By reshaping statutory quality standards for end products along the industrial chain, The Consistency Evaluation Policy of Generic Drugs transmits institutional pressure back to the R&D stage. Enterprises are therefore required to make substantive technical improvements in generic-drug formulations, crystal forms, and process parameters. These improvements are needed to address a key technical challenge in large-scale production: achieving a high degree of consistency between *in vitro* dissolution and *in vivo* bioequivalence (BE). To pass the evaluation, enterprises are compelled to strengthen applied research and industrialization capabilities at the downstream end of the innovation chain (16). In the short term, the consistency evaluation may lead enterprises to allocate more resources toward high-quality generic drug development and incremental improvements, thereby exerting some crowding-out on original innovation. However, in the long run, the policy eliminates low-end production capacity and forces enterprises to establish a solid R&D system. It also helps accumulate talent, technology, and funding for the innovation of original drugs, continuously strengthening the micro-foundations of the innovation chain (17, 18).

Positive optimization of the industrial chain structure through consistency evaluation. The policy directly accelerates the elimination of low-end production capacity. Drugs that fail to pass the evaluation are required to be withdrawn from the market. This reduces excessive and redundant approvals for the same product varieties and creates market space for high-quality products (6). Drugs that pass the evaluation receive official endorsement of their clinical and economic values. They therefore gain significant advantages in bidding procurement and medical insurance payment (3), which prompts more efficient concentration of resources toward high-quality enterprises. More importantly, the policy reshapes the competitive logic of the industrial chain. Competition shifts away from destructive rivalry driven by channels and price. It moves toward healthier competition centered on product quality and brand.

During the integration of the innovation chain and the industrial chain, consistency evaluation plays a pivotal bridging role. It provides a unified and authoritative standard and a shared basis for coordination. The policy requires that R&D design be closely aligned with process scale-up and quality control capabilities. This encourages enterprises to adopt the Quality by Design (QbD) approach and move away from an isolated laboratory model. From the outset of a project, enterprises must consider process robustness, cost efficiency, and dissolution-curve consistency. This supports a shift from laboratory feasibility to industrial equivalence. Simultaneously, to meet evaluation standards, enterprises along the industrial chain must upgrade equipment, optimize processes, and strengthen supply chain management in reverse. The resulting feedback information can directly guide the iterative direction of the innovation chain (19). As a powerful quality supervision tool, the consistency evaluation policy improves the quality of generic drugs at a critical node. It pulls the innovation chain to become more solid and refined, and it positively optimizes the industrial chain structure. Under the market mechanism of survival of the fittest, the policy promotes the integrated development of the innovation chain and industrial chain in the pharmaceutical manufacturing industry. Based on this analysis, research hypothesis 1 (H1) is proposed:

*H1*: The Consistency Evaluation Policy of Generic Drugs can significantly promote the integrated development of the innovation chain and industrial chain in the pharmaceutical manufacturing industry.

### 2.2 The mechanism of the consistency evaluation policy on the integration of innovation chain and industrial chain in the pharmaceutical manufacturing industry

As a mandatory market-access standard, the Consistency Evaluation Policy of Generic Drugs represents a supply-side reform aimed at improving medication quality for the population. Its implementation has prompted both central and local governments to introduce fiscal subsidy programs. This is mainly because the policy carries substantial strategic significance and markedly increases enterprises’ costs of research and development and quality upgrading. Government intervention is therefore needed to mitigate transitional pressures and support effective policy implementation. On the one hand, subsidies serve as a crucial economic lever for the government to guide industrial upgrading and can reinforce integrated innovation along the industrial chain (20, 21). The government, through special fund subsidies, precisely supports the evaluation of clinically essential and market-shortage drug varieties, bridging the key links between R&D translation and market application. This facilitates the rapid integration of innovative achievements into the production system, thereby enhancing industrial chain security. Concurrently, “post-subsidies” are used to incentivize enterprises that are the first to pass the evaluation, guiding the transmission of quality standards upstream and downstream. This promotes the transformation of technological advantages into industrial advantages, accelerates the agglomeration of innovation factors in high-quality enterprises, and ultimately achieves the deep integration of the innovation chain and industrial chain. On the other hand, consistency evaluation has sharply increased industry costs, and the evaluation cost for a single product can reach several million to tens of millions of yuan (22). If enterprises bear these costs entirely on their own, many small and medium-sized pharmaceutical companies may relinquish their drug approval numbers due to financial constraints. This could lead to supply shortages for some basic medicines, thereby affecting industrial chain stability and drug accessibility. Subsidies can therefore share enterprises’ costs and provide a buffer for industrial upgrading. They can also help mitigate front-end R&D risks. As a result, enterprises may allocate more internal resources to innovative drugs and high-end generics. This mechanism promotes the extension of resources from foundational stages like consistency evaluation to the front-end of R&D and innovation, fostering the synergy between technological accumulation and industrial upgrading. It accelerates the formation of an industrial system driven by innovation and oriented by clinical needs, promoting the comprehensive integration of innovation elements and industrial resources. Based on analysis, research hypothesis 2 (H2) is proposed:

*H2*: The Consistency Evaluation Policy of Generic Drugs promotes the integrated development of the innovation chain and industrial chain in the pharmaceutical manufacturing industry by increasing government subsidies.

As a significant reform policy in China’s pharmaceutical sector, consistency evaluation accelerates survival of the fittest and significantly increases industry concentration (6), thereby promoting the transformation of market structure toward intensification and large-scale development. The impact of consistency evaluation on market concentration is mainly reflected in three aspects: First, a large amount of outdated production capacity is directly eliminated. Consistency evaluation requires generic drug varieties to pass the evaluation within a specified time frame; otherwise, their approval numbers will be revoked. The high cost of evaluating individual varieties compels numerous small and medium-sized enterprises with weak financial and technological capabilities to voluntarily relinquish their approval numbers, directly resulting in a sharp decline in the number of market participants. Second, it reshapes competition rules and raises industry barriers. By directly linking consistency evaluation with market access and medical insurance reimbursement (23, 24), leading enterprises intensify investment and rapidly expand market share after passing the evaluation, resulting in a “Matthew effect” in which stronger enterprises become stronger. Third, it promotes horizontal mergers, acquisitions, and integration among pharmaceutical companies. Assets such as approval numbers and production lines of enterprises that fail the evaluation become acquisition targets, allowing leading enterprises to broaden product portfolios and expand market share, further consolidating market positions.

As consistency evaluation progresses, industrial resources and market shares tend to concentrate among enterprises with strong innovative-drug R&D capabilities and technological advantages in producing high-quality generics. This contributes to a sustained increase in market concentration (18). At the entity level, dominant enterprises leverage their economies of scale to strengthen vertical integration capabilities. By extending into the upstream supply chain, they ensure a stable supply of innovation resources, laying the foundation for sustained investment in high-risk R&D. At the ecological level, increased market concentration reduces the costs and complexity of inter-enterprise collaboration, facilitating the formation of stable and efficient innovation cooperation networks. This drives innovation activities from within individual enterprises to the entire industrial landscape, thereby achieving the deep integration of the innovation chain and industrial chain. Based on this analysis, research hypothesis 3 (H3) is proposed:

*H3*: The Consistency Evaluation Policy of Generic Drugs promotes the integrated development of the innovation chain and industrial chain in the pharmaceutical manufacturing industry by enhancing market concentration.

## 3 Indicator measurement and research design

### 3.1 Measurement of the integration of innovation chain and industrial chain

Enterprises are the crucial carriers of knowledge creation along the innovation chain and value realization along the industrial chain. The depth of their activities and operational efficiency directly reflect the actual level of integration between the dual chains. This paper is grounded in the micro-foundations of macro concepts, regards enterprises as the fundamental components of both the innovation chain and the industrial chain. From the perspective of nodal enterprises, the study constructs an evaluation index system for the integration of the innovation chain and industrial chain applicable to the pharmaceutical manufacturing industry. Specifically, we use the entropy-weight method to measure the levels of the innovation chain and the industrial chain, respectively. Then the coupling coordination degree model is utilized to calculate the coupling coordination degree between the innovation chain and the industry chain in the pharmaceutical manufacturing industry (25).

Indicators for the innovation chain. The pharmaceutical manufacturing industry is a typical research and development–driven sector, and the innovation chain can be assessed through a structured set of indicators. First, R&D expenditure intensity captures enterprises’ allocation of resources to R&D activities and reflects their willingness to innovate, providing a baseline condition for sustained innovation. Second, R&D personnel input reflects enterprises’ support for innovation in human capital; core technical teams are especially important for breakthrough innovation. Third, we measure innovation outputs using patent-based indicators. The number of granted patents, particularly invention patents, reflects the volume of innovation outcomes. The average citation frequency of patents captures impact and diffusion, serving as a proxy for innovation quality and industry recognition. Fourth, clinical trials represent a distinctive translational step in this field. The number of registered clinical trials reflects the breadth of a enterprise’s R&D pipeline and the intensity of translational activity. Finally, enterprise value in this industry relies heavily on intangible assets such as patents and technologies. We therefore use the share of intangible assets in total assets to measure the capitalization of innovation outcomes. This indicator captures both the financial value created by innovation and the pathway through which enterprises build competitive advantage.

Indicators for the industrial chain. From a supply-chain perspective, upstream concentration is a core indicator of supply security, cost control, and technological autonomy. Diversifying supplier sources can reduce the risk of raw-material disruptions. It can also enhance bargaining power and lower dependence on specific external technologies. Downstream concentration reflects revenue stability and market influence. It also captures sensitivity to changes in major customers or policy adjustments. A more diversified customer base strengthens the robustness of revenue sources. It helps preserve pricing power and profit margins. It also cushions shocks from policy measures such as volume-based procurement. From an asset-structure perspective, employee scale captures organizational complexity and operating scale. Fixed-asset scale reflects production capacity and capital barriers. Production facilities that meet Good Manufacturing Practice (GMP) standards constitute a basic entry requirement in the industry. Accordingly, fixed-asset investment also signals a enterprise’s comprehensive strength. Finally, cost control and profitability are key outcome indicators for assessing industrial-chain positioning and asset allocation. Under volume-based procurement, which trades lower prices for higher volumes, strong cost control provides a buffer against price pressure and policy risk. Moreover, this is a capital-intensive and R&D-intensive industry. Sound profitability reflects the efficiency of asset allocation. It also provides stable financial support for sustained R&D investment and high-risk innovation projects. This forms the basis of long-term competitiveness. Table 1 Evaluation indicators for the innovation chain and industrial chain in the pharmaceutical manufacturing industry.

**Table 1:** Evaluation indicators for the innovation chain and industrial chain in the pharmaceutical manufacturing industry.

Chain	Indicator	Indicator calculation process
Innovation chain	R&D expenditure	R&D expenses/operating revenues
	R&D personnel input	Number of technical personnel/Total number of employees
	Innovation capability	Number of invention patent grants
	Innovation quality	Average number of patent citations
	Clinical trial translation	Number of clinical trial registrations
	Excess profitability	Intangible assets value/total assets
Industrial chain	Upstream concentration	Ratio of procurement amounts from the top five suppliers to the total annual procurement amount
	Personnel input	Number of current employees
	Fixed asset investment	Net value of fixed assets
	Cost control capability	Operating profit/operating cost
	Profitability	Net profit/total assets
	Downstream concentration	Ratio of sales from top five customers to total annual sales

### 3.2 Model setting

We treat the Opinions on Conducting the Quality and Efficacy Consistency Evaluation of Generic Drugs, issued by the General Office of the State Council on March 5, 2016 (26), as a quasi-natural experiment. We apply Difference-in-Differences (DID) method to evaluate the impact of the Consistency Evaluation Policy of Generic Drugs on the integration of the innovation chain and industrial chain in the pharmaceutical manufacturing industry. The empirical model is set up as follows:


$$D_{\mathrm{it}}=\beta_{0}+\beta_{1}{\text{treat}}_{i}\times {\text{post}}_{t}+\beta_{2}X_{\mathrm{it}}+\mu_{i}+\theta_{t}+\varepsilon_{\mathrm{it}}$$ (1)

In Equation (1), *i* and *t* represent enterprises and years, respectively. The dependent variable $D_{\mathrm{it}}$ denotes the degree of dual chains integration. $\text{trea}t_{i}$ and $\mathrm{pos}t_{t}$ are dummy variables. $\text{trea}t_{i}=1$ when a enterprise belongs to the chemical preparation industry and $\text{trea}t_{i}=0$ otherwise; $\mathrm{pos}t_{t}=1$ when the year is 2016 or later and $\mathrm{pos}t_{t}=0$ otherwise. $X_{\mathrm{it}}$ is a vector of control variables. $\mu_{i}$ represents enterprise fixed effects. $\theta_{t}$ represents year fixed effects, and $\varepsilon_{\mathrm{it}}$ is the random error term.

### 3.3 Variable descriptions

#### 3.3.1 Dependent variable

The dependent variable is the degree of integration between the innovation chain and the industrial chain in the pharmaceutical manufacturing industry. We measure it using the coupling coordination degree ( $D_{\mathrm{it}}$ ) between dual chains.

#### 3.3.2 Explanatory variable

The key explanatory variable is the interaction term between the policy dummy variable and the time dummy variable. Because the consistency evaluation of the quality and efficacy of generic drugs is aimed at chemical drugs, it is not directly applicable to non-chemical drugs (such as traditional Chinese medicine, biological products, etc.). Therefore, this paper uses a policy dummy variable (treat) of “industries to which the consistency evaluation applies or not” to measure the consistency evaluation policy. If a listed enterprise belongs to the chemical preparation industry, it is assigned a value of 1; if it belongs to non-chemical preparation industries, it is assigned a value of 0. We identify chemical preparation enterprises using the Shenwan Level-3 industry classification and enterprises’ industry codes (27, 28). Enterprises in the chemical preparation industry form the treatment group, while other enterprises form the control group. For the time dimension, we set 2016 as the policy implementation year. When the year in question is 2016 or later, the value of post is set to 1; otherwise, it is set to 0.

#### 3.3.3 Control variables

To mitigate omitted-variable bias, we include the following controls: enterprise size (size), measured as the natural logarithm of total assets; enterprise digital transformation (Dig), measured by the digital transformation index from the CSMAR database; ownership type (soe), equal to 1 for state-controlled enterprises and 0 otherwise; administrative expense ratio (admin), measured as administrative expenses divided by main business revenue; selling expense ratio (ser), measured as selling expenses divided by main business revenue; ownership concentration (Top5), measured by the shareholding ratio of the top five shareholders; and executive compensation incentive (Pay), measured as the natural logarithm of total annual compensation of the management team.

### 3.4 Data description

Our sample consists of pharmaceutical manufacturing enterprises listed on China’s Shanghai and Shenzhen A-share markets from 2013 to 2023. The main data sources are the CSMAR database and the China National Research Data Services Platform (CNRDS). We compile the number of clinical trial registrations from the Drug Clinical Trial Registration and Information Disclosure Platform. And the initial samples are processed as follows: we exclude enterprises marked as ST, *ST, or PT; we exclude enterprises with industry codes other than C27; we drop observations with severe financial abnormalities or missing data; and we exclude enterprises with a sample period of less than 3 years. The final sample includes 285 enterprises and 2,248 enterprise-year observations over 2013–2023.

## 4 Empirical results and analysis

### 4.1 Benchmark regression results

Table 2 presents the benchmark regression results of this paper. In the regression of column (1), only the key explanatory variable, enterprise fixed effects, and year fixed effects are included. The estimated coefficient on the Consistency Evaluation Policy of Generic Drugs is positive and statistically significant at the 1% level. Column (2) presents the regression results after further incorporating other control variables. The results remain robust. The policy coefficient is still significantly positive, with an estimated value of 0.022, and it is significant at the 1% level. In economic terms, the policy has led to an average increase of 2.2 percentage points in the level of dual chains integration among the sample enterprises. These findings indicate that The Consistency Evaluation Policy of Generic Drugs significantly promotes the integration of the innovation chain and the industrial chain in the pharmaceutical manufacturing industry, thereby supporting Hypothesis 1 (H1).

**Table 2:** The impact of the consistency evaluation policy of generic drugs on the integration of the innovation chain and the industrial chain in the pharmaceutical manufacturing industry.

Variables	(1) *D*	(2) *D*
did	0.019** (0.008)	0.022*** (0.007)
size	—	0.026*** (0.004)
dig	—	0.001** (0.000)
soe	—	−0.016** (0.007)
admin	—	0.027*** (0.009)
ser	—	−0.016* (0.010)
Top5	—	0.041** (0.020)
Pay	—	0.002 (0.002)
Constant	0.234*** (0.002)	−0.422*** (0.091)
Firm FE	YES	YES
Year FE	YES	YES
Observations	2,248	2,248
*R* ^2^	0.871	0.887

***, **, and * indicate significance at the 1, 5, and 10%, respectively. Cluster-robust standard errors are reported in parentheses. The same convention applies to the tables below.

### 4.2 Robustness tests

#### 4.2.1 Parallel trend test

**Figure 1:**
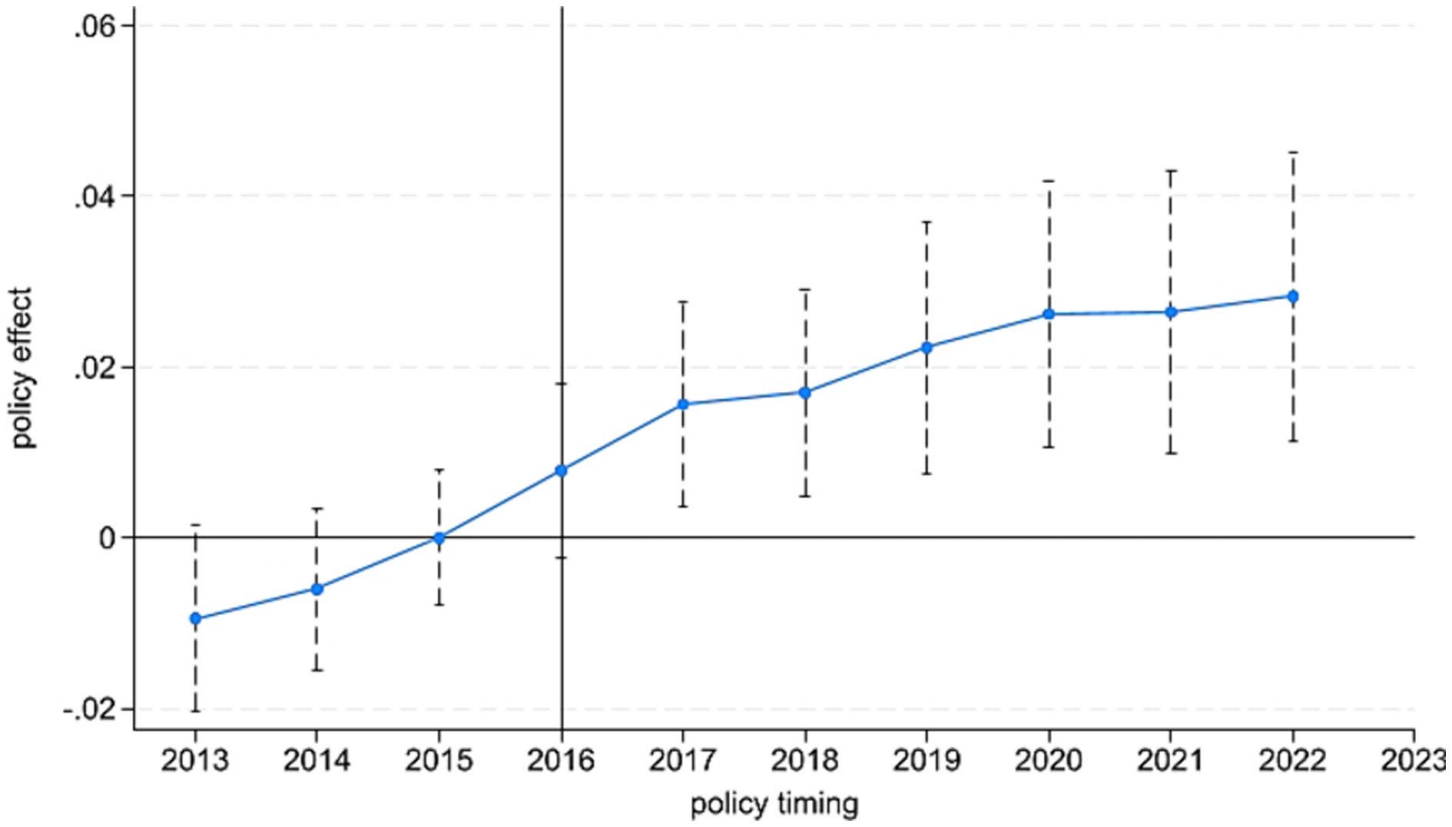
Parallel trend test.

The DID design relies on a key identifying assumption: prior to the policy intervention, the treatment and control groups should follow a common time trend in the outcome variable. This assumption provides the basis for identifying the net policy effect (29, 30). Following the event-study approach, we conduct a parallel trend test using 2016 as the policy implementation year. Figure 1 reports the results. Before 2016, the estimated coefficients are not statistically significant at the 5% level, indicating no systematic pre-policy differences between the treatment and control groups. This supports the parallel-trend assumption. After the policy shock, the coefficients become significantly positive and exhibit an upward pattern, suggesting a marked increase in the integration of the innovation chain and the industrial chain in the pharmaceutical manufacturing industry. These results imply that the Consistency Evaluation Policy of Generic Drugs has a meaningful effect in promoting dual chains integration.

#### 4.2.2 Placebo test

**Figure 2:**
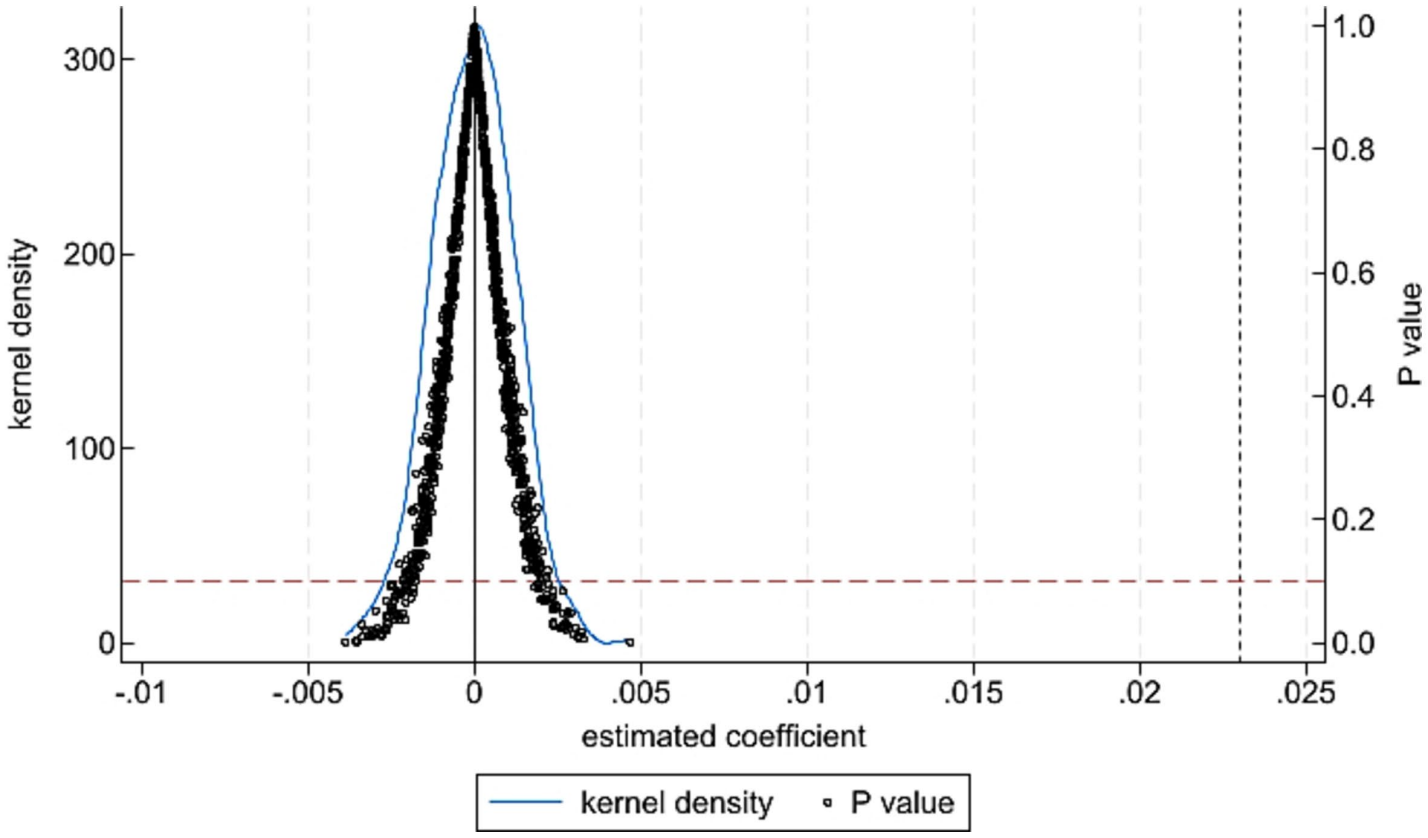
Placebo test.

To rule out the possibility that the estimated policy effect is driven by unobservable random factors, we conduct a placebo (permutation) test (31). If the benchmark result were spurious, the estimated coefficient would be centered around zero and statistically insignificant when the policy assignment is randomly reassigned. We perform 1,000 random reassignment experiments and plot the kernel density distribution of the resulting coefficients (Figure 2). The placebo coefficients are concentrated around zero and are substantially smaller than the benchmark estimate (0.022). In addition, the distribution is approximately normal, and most *p*-values exceed 0.100, indicating insignificance at the 10% level. These results suggest that the observed effect of the Consistency Evaluation Policy of Generic Drugs on dual chains integration is unlikely to be driven by random factors. The benchmark findings are therefore robust.

#### 4.2.3 Endogeneity test

##### 4.2.3.1 Heckman two-stage method

To alleviate the endogeneity issues caused by potential sample selection bias in the progressive DID fixed-effects model, this study draws on existing research methods and constructs a Heckman two-stage selection model for handling. In the first stage, we implement a Heckman two-stage selection model. In the first stage, we estimate a Probit model where the dependent variable indicates whether a enterprise belongs to the chemical preparation industry (1 if yes, 0 otherwise). The covariates include enterprise size (*size*), digital transformation (*Dig*), ownership type (*soe*), administrative expense ratio (*admin*), selling expense ratio (*ser*), ownership concentration (*Top5*), and executive compensation incentive (*Pay*). Based on the first-stage estimates, we compute the inverse Mills ratio (IMR) to capture selection bias. In the second stage, we include the IMR in the baseline regression and re-estimate the effect of the Consistency Evaluation Policy of Generic Drugs on dual chains integration. The results in Column (1) of Table 3 show that, after controlling for sample selection bias, the estimated coefficient of the key explanatory variable is significantly positive, indicating that the consistency evaluation policy can significantly promote the integration of the dual chains. This indicates that the policy effect is not driven by sample self-selection. Overall, the Heckman results are consistent with the benchmark regression, supporting the robustness of our main findings.

**Table 3:** Endogeneity test.

Variables	(1) Heckman two-stage	(2) PSM-DID
did	0.0217^***^ (3.13)	0.021*** (0.008)
IMR	−0.3631 (−1.57)	—
Control variables	YES	YES
Firm FE	YES	YES
Year FE	YES	YES
Observations	2,248	1,484
*R* ^2^	0.8871	0.901
Adjust-*R*^2^	0.8695	0.8802

##### 4.2.3.2 PSM-DID method

We further assess robustness using the propensity score matching-difference-in-differences method (PSM-DID) (32). Using enterprise size (*size*), enterprise digital transformation (*Dig*), enterprise ownership nature (*soe*), administrative expense ratio (*admin*), proportion of selling expenses (*ser*), equity concentration (*Top5*), and executive compensation incentives (*Pay*) as matching covariates. We then conduct 1:1 nearest-neighbor matching. After conducting regression analysis again with the matched samples, the regression results in column (2) of Table 3 show that the estimated coefficient of the consistency evaluation policy on the integration of the dual chains is significantly positive, indicating that this policy can promote the integration of the dual chains. The above analysis demonstrates that, after controlling for sample selection bias, the empirical results are highly consistent with the benchmark conclusions, further confirming the robustness of the research findings.

#### 4.2.4 Replacing the measurement method of the explained variable

First, we use a modified coupling coordination model to measure the integration of the innovation chain and the industrial chain (25). As shown in column (1) of Table 4, the estimated coefficient of the consistency evaluation policy is 0.024, and the regression result remains significant at the 1% level. Second, measure the integration of innovation chain and industrial chain by referring to the composite system coordination degree model (33). As shown in column (2) of Table 4, the estimated coefficient for the consistency evaluation policy is 0.013, which has passed the 1% significance test. Overall, the policy effect remains positive and statistically significant after replacing the measurement of the dependent variable. This supports the robustness of the benchmark results.

**Table 4:** Robustness tests.

Variables	(1) Replace the measurement method of the explained variables	(2) Replace the measurement method of the explained variables	(3) Winsorization	(4) Lagging the explanatory variables by one period
did	0.024*** (0.007)	0.013*** (0.004)	0.017*** (0.005)	—
L.did	—	—	—	0.024*** (0.007)
Control variables	YES	YES	YES	YES
Firm FE	YES	YES	YES	YES
Year FE	YES	YES	YES	YES
Observations	2,248	2,248	2,248	1958
*R* ^2^	0.797	0.816	0.892	0.902

#### 4.2.5 Sample winsorization treatment

Considering that extreme values and abnormal observations may affect the empirical results, we conduct 1 and 99% winsorization on the original data and then perform regression analysis. The regression results in Column (3) of Table 4 show that after winsorization, the positive impact of the consistency evaluation policy on the integration of the innovation chain and industrial chain in the biomedical industry remains significant at the 1% level. This indicates that the benchmark findings are not driven by extreme observations.

#### 4.2.6 Lagging the explanatory variables by one period

The policy effect may materialize with a lag. We therefore re-estimate the model using a one-period lag of the key explanatory variable. The regression results in Column (4) of Table 4 show that the estimated coefficient of the consistency evaluation policy lagged by one period is 0.024, which remains significant at the 1% level. The magnitude is broadly consistent with the benchmark results in Table 2. This demonstrates that the consistency evaluation policy promotes the integration of the innovation chain and industrial chain in the pharmaceutical manufacturing industry and exhibits good robustness.

### 4.3 Mechanism test

The Consistency Evaluation Policy of Generic Drugs may promote the integration of the innovation chain and industrial chain in the pharmaceutical manufacturing industry by increasing government subsidies received by enterprises and enhancing market concentration. Following the approach of Rajan and Zingales (34), we test these mechanisms using an interaction-term specification. If the policy operates through these channels, it should generate a stronger integration effect for enterprises that initially receive higher government subsidies or operate in more concentrated markets. For such enterprises facing the consistency evaluation policy, their role in promoting the integrated development of the innovation chain and industrial chain will be more pronounced (35). We therefore estimate the following model to examine whether the policy promotes dual chains integration by increasing government subsidies and market concentration.


$$D_{\mathrm{it}}=\beta_{0}+\beta_{1}u_{\mathrm{it}}\times \mathrm{di}d_{\mathrm{it}}+\beta_{2}u_{\mathrm{it}}+\beta_{3}\mathrm{di}d_{\mathrm{it}}+\beta_{4}X_{\mathrm{it}}+\mu_{i}+\theta_{t}+\varepsilon_{\mathrm{it}}$$ (2)

In Equation (2), $\;u_{\mathrm{it}}\;$ includes two variables: government subsidies (subsidy) and market concentration (*HHI*). If the consistency evaluation policy indeed helps to increase the government subsidies received by pharmaceutical manufacturing enterprises and enhance market concentration, thereby promoting the integrated development of the innovation chain and industrial chain, then the coefficient $\beta_{1}$ of the interaction term should be significantly positive.

#### 4.3.1 Effects of government subsidies

The Consistency Evaluation Policy of Generic Drugs has significantly raised the market entry standards for the pharmaceutical manufacturing industry, prompting enterprises to increase their R&D investment to pass the rigorous evaluations of drug quality and efficacy. To this end, governments at all levels have provided special government subsidies to share the high evaluation costs incurred by enterprises and guide industrial upgrading and the supply of high-quality drugs. On the one hand, government subsidy funds are closely linked to the successful passing of evaluations, incentivizing enterprises to strive for successful evaluation outcomes. Then it can alleviate their financial pressure and risks associated with imitation. On the other hand, this approach strengthens the policy-guiding role of subsidy funds by prioritizing support for clinically urgent and market-short varieties, as well as generics with high technical difficulty. Next, it can encourage enterprises to conduct evaluations with clinical value, optimize their product mix, eliminate redundant and outdated production capacities, and ultimately drive the entire industry toward high-quality innovation and development. We measure subsidy as the natural logarithm of the amount of government subsidies received by listed enterprises. The analysis results are shown in column (1) of Table 5. The estimated coefficient of the interaction term did×subsidy is significantly positive, indicating that for enterprises with higher initial government subsidies, the consistency evaluation policy can more effectively increase their government subsidy amounts and promote a more pronounced effect on the integration and development of the innovation chain and industrial chain. Hypothesis 2 (H2) is supported.

**Table 5:** Mechanism test.

Variables	(1) Government subsidies	(2) Market concentration
did×subsidy	0.010*** (0.003)	—
did×HHI	—	0.571** (0.249)
Control variables	YES	YES
Firm FE	YES	YES
Year FE	YES	YES
Observations	2,248	2,248
*R* ^2^	0.890	0.887

#### 4.3.2 Market concentration effect

The Consistency Evaluation Policy of Generic Drugs, as a core measure of supply-side structural reform in the pharmaceutical manufacturing industry, has significantly raised the industry’s technological and financial barriers. Small and medium-sized enterprises, unable to bear the exorbitant evaluation costs and research and development risks, are compelled to abandon some approval numbers or even exit the market. Conversely, leading enterprises, leveraging their strong R&D capabilities and substantial financial resources, can efficiently complete evaluations for multiple product varieties and seize the market share vacated by those exiting enterprises. The result is an acceleration of the survival-of-the-fittest process in the industry, driving a rapid concentration of market share toward leading enterprises and significantly enhancing the market concentration in the pharmaceutical manufacturing sector. We measure market concentration of enterprises using the Herfindahl–Hirschman Index (HHI). The analysis results are shown in column (2) of Table 5. The estimated coefficient of the interaction term did×HHI is significantly positive, indicating that the higher the market concentration of enterprises, the more the consistency evaluation policy can enhance the market concentration of such enterprises, thereby promoting the integrated development of the innovation chain and industrial chain in the pharmaceutical manufacturing industry. Hypothesis 3 (H3) is supported.

### 4.4 Heterogeneity analysis

#### 4.4.1 Market demand scale

Market demand serves as the core orientation and fundamental driving force that leads the collaborative development of the innovation chain and industrial chain in the pharmaceutical manufacturing industry. A larger demand scale implies higher expected returns. These returns can better cover the substantial costs associated with the evaluation process. Enterprises therefore have stronger capacity to conduct pharmaceutical studies and bioequivalence tests required to pass the evaluation. This process also pushes enterprises to upgrade their R&D capabilities and quality management systems. After passing the evaluation, winning bids in volume-based procurement can secure relatively stable market shares. The resulting profits can, in turn, support subsequent innovative R&D and promote high-end and refined upgrading of the industrial chain. Following Wang and Tao (36), we split the sample into enterprises with larger versus smaller market demand scales using the median of operating revenue and estimate group-specific regressions. Columns (1) and (2) of Table 6 report the results. The estimated coefficients are significantly positive in both subsamples. However, the coefficient is larger for enterprises with a larger demand scale, and the difference passes the coefficient-difference test. This suggests that under The Consistency Evaluation Policy of Generic Drugs, enterprises with larger market demand play a more prominent role in promoting integration between the innovation chain and the industrial chain.

**Table 6:** Heterogeneity analysis.

Variables	(1) Large market demand scale	(2) Small market demand scale	(3) High TFP	(4) Low TFP
Did	0.030*** (0.011)	0.010** (0.005)	0.036*** (0.013)	0.005 (0.005)
Control variables	YES	YES	YES	YES
Firm FE	YES	YES	YES	YES
Year FE	YES	YES	YES	YES
Observations	1,112	1,107	1,103	1,086
*R* ^2^	0.886	0.737	0.897	0.791
Coefficient difference	−0.02***		−0.032***	
*p*-value for coefficient difference test	0.009		0.000	

The “empirical *p*-value” tests whether the DID coefficients differ across groups. It is based on Fisher’s combination test and is obtained from 1,000 bootstrap resamples.

#### 4.4.2 Total factor productivity of enterprises

Enterprise total factor productivity (TFP) serves as the critical support for driving the deep integration of the innovation chain and industrial chain. For enterprises with high TFP, their advanced research and development, production, and quality management systems mean that the consistency evaluation policy is not a burden but rather a catalyst for leveraging their competitive advantages. These enterprises can efficiently pass the evaluations and swiftly introduce drugs that meet the standards into the market, seizing shares of original drugs. The policy also encourages faster translation of innovation outputs into high-quality products. Scale economies can further reduce costs. Together, these mechanisms strengthen an efficient loop from innovative R&D to industrialization and marketization, and they reinforce deeper integration between the dual chains. We measure enterprise TFP following Lu and Lian (37) and split the sample into higher-TFP and lower-TFP groups using the median value. Columns (3) and (4) of Table 6 present the results. For the higher-TFP group, the estimated coefficient is 0.036 and is significant at the 1% level. For the lower-TFP group, the coefficient is positive but not statistically significant. This indicates that the policy is more effective in promoting dual chains integration among higher-TFP enterprises in the pharmaceutical manufacturing industry.

## 5 Conclusion and policy implications

### 5.1 Conclusion

Using data on China’s A-share listed pharmaceutical enterprises from 2013 to 2023, this study examines the effects and mechanisms of The Consistency Evaluation Policy of Generic Drugs on integration between the innovation chain and the industrial chain in the pharmaceutical manufacturing industry. The main conclusions are as follows.

(1) The consistency evaluation policy can promote the integrated development of the innovation chain and industrial chain in the pharmaceutical manufacturing industry, and this conclusion remains valid after a series of robustness tests, including addressing endogeneity issues and replacing variable indicators.
(2) The consistency evaluation policy drives the integrated development of the innovation chain and industrial chain in the pharmaceutical manufacturing industry through two channels: government subsidies and increased market concentration.
(3) Compared with enterprises with a small market demand scale, those with a large market demand scale play a more significant role in promoting the integration of the innovation chain and the industrial chain. For enterprises with high total factor productivity, the consistency evaluation policy significantly promotes the integration of the innovation chain and the industrial chain in the pharmaceutical manufacturing industry, while its impact effect is not significant for enterprises with low total factor productivity.

### 5.2 Policy recommendations

First, continuously optimize the depth and precision of consistency evaluation. By dynamically raising standards and deeply integrating evaluation results with backend links such as government procurement, medical insurance payment, and clinical application, a market reward mechanism of “high quality, favorable price” can be established to drive enterprises to continuously invest in R&D and process improvement. Focus on supporting leading enterprises that have passed the evaluation to play the role of “chain leaders” and take the lead in forming innovation consortia. At the same time, assist “specialized, refined, distinctive, and innovative” small and medium-sized enterprises in integrating into the industrial chain, achieving integrated innovation and collaborative development among large, medium, and small enterprises.

Second, precisely utilize government subsidies to guide and enhance market concentration by leveraging the situation. Government subsidies should be primarily directed toward high-quality varieties that emerge from consistency evaluations, key technology research and development, as well as critical links connecting the innovation chain with the industrial chain, thereby improving the efficiency of fund utilization. Support can also be provided to compliant leading enterprises to conduct cross-regional and cross-ownership mergers and acquisitions. Such restructuring can help integrate resources, phase out outdated capacity, and foster internationally competitive leading enterprises and specialized industrial clusters.

Third, implement differentiated guidance strategies and adopt multiple measures to enhance enterprise vitality. For leading enterprises, encourage them to establish open innovation platforms and take the lead in forming full-chain innovation consortia covering “drug discovery - clinical development – industrialization,” thereby driving upgrading and integration across the industrial chain. For smaller enterprises with specific technological advantages, guide them to focus on technological innovation and process improvement in featured active pharmaceutical ingredients and niche fields, enabling them to become indispensable “specialized, refined, distinctive, and innovative” links in the industrial chain. Enterprises can also be encouraged to use the evaluation process as an opportunity to advance smart and green upgrading of production lines. Digital transformation can improve production efficiency and product quality stability. Finally, cultivating interdisciplinary talent with expertise in both pharmaceutical R&D and industrialization can support smoother translation of innovation outputs and ongoing optimization of the industrial chain.

## Data Availability

The original contributions presented in the study are included in the article/supplementary material, further inquiries can be directed to the corresponding author.
